# Compatibility between mitochondrial and nuclear genomes correlates with the quantitative trait of lifespan in *Caenorhabditis elegans*

**DOI:** 10.1038/srep17303

**Published:** 2015-11-25

**Authors:** Zuobin Zhu, Qing Lu, Fangfang Zeng, Junjing Wang, Shi Huang

**Affiliations:** 1 State Key Laboratory of Medical Genetics, School of Life Sciences, Xiangya Medical School, Central South University, 110 Xiangya Road, Changsha, Hunan, 410078, China

## Abstract

Mutations in mitochondrial genome have epistatic effects on organisms depending on the nuclear background, but a role for the compatibility of mitochondrial-nuclear genomes (mit-n) in the quantitative nature of a complex trait remains unexplored. We studied a panel of recombinant inbred advanced intercrossed lines (RIAILs) of *C. elegans* that were established from a cross between the N2 and HW strains. We determined the HW nuclear genome content and the mitochondrial type (HW or N2) of each RIAIL strain. We found that the degree of mit-n compatibility was correlated with the lifespans but not the foraging behaviors of RIAILs. Several known aging-associated QTLs individually showed no relationship with mitotypes but collectively a weak trend consistent with a role in mit-n compatibility. By association mapping, we identified 293 SNPs that showed linkage with lifespan and a relationship with mitotypes consistent with a role in mit-n compatibility. We further found an association between mit-n compatibility and several functional characteristics of mitochondria as well as the expressions of genes involved in the respiratory oxidation pathway. The results provide the first evidence implicating mit-n compatibility in the quantitative nature of a complex trait, and may be informative to certain evolutionary puzzles on hybrids.

Lifespans of individuals in a population are well known to be a complex quantitative trait, the genetic component of which is usually explained by nuclear genome variations such as single nucleotide polymorphisms^1^,^2^,^3^. Mitochondria are indispensable for aerobic life. Depending on the tissue and species, they normally occupy 2–20% of the volume of a cell^4^. Accumulating evidence has suggested a link between mitochondrial dysfunction or variants and aging^5^,^6^,^7^. Mitochondria free radical theory of aging suggests that the main cause of aging is the accumulation of damage resulting from the mitochondrial production of toxic reactive oxygen species (ROS)^8^. A large number of studies have shown that increased ROS production and oxidative damage can influence aging related phenotypes or diseases^9^,^10^,^11^,^12^. But there were also findings showing ROS as an important signaling molecule that can actually have beneficial effects on longevity^13^,^14^,^15^. Recent studies have implicated in aging a mitochondrial stress response, the mitochondrial unfolded protein response^16^. These studies indicate that multiple elements of mitochondria are involved in aging.

There are over 1000 proteins associated with the function of mitochondria and only 13 are encoded by the mitochondrial DNA (mtDNA), while the others are encoded by the nuclear genome and imported into mitochondria. A significant fraction of nuclear genes is involved in aging, e.g., in *C. elegans*, there are almost 900 genes with an eQTL, of which almost half were found to have a genotype-by-age effect^17^. Recently, mitochondrial-nuclear(mit-n) interaction in fitness has been studied in many species^18^,^19^,^20^,^21^. There are several lines of evidence for co-evolution of mitochondrial and nuclear genomes^22^,^23^,^24^,^25^.

When compared with N2 wild isolates, the Hawaii (HW) wild isolates had a unique p.A12S amino acid substitution in the mtDNA-encoded COX1 core catalytic subunit of mitochondrial complex IV^26^. A hybrid strain of *C. elegans* with the HW mitochondria in the N2 nuclear background had reduced median lifespan relative to the HW strain CB4856^26^. While this result may be related to nuclear–mtDNA mismatch, it remains unknown whether this mismatch involves mainly certain specific loci such as the p.A12S amino acid or the whole set of mitochondrial and nuclear genome. Also unclear is whether the quantitative trait of aging in a population is related to the quantitative variations in the compatibility between mitochondria and nuclear genomes.

*C. elegans* as a model for longevity research has been widely studied^17^,^27^,^28^,^29^. Several QTLs linked with lifespan of *C. elegans* have been found using a genome-wide library of CB4856/N2 introgression lines^30^. We here studied the recombinant inbred advanced intercross lines (RIAILs) of *C. elegans*, which were derived from the laboratory strain N2 (Bristol strain) and the natural isolate CB4856 (Hawaii strain)^31^. There were great genetic variations in the two strains in both the nuclear^32^ and the mitochondrial genomes^26^. In generating these RIAILs, the F1 up to F10 progenies were intercrossed to maximize random recombination and hence allelic diversity in the offspring population, which were then randomly selected for inbreeding upto to 20 generations to generate the final panel of strains. There were abundant recombination between the nuclear genomes of the N2 and HW strains and between the nuclear and mitotypes of these strains. Because of the random recombination between males and hermaphrodites, the mitotypes of the strains were indeterminated^31^.

Here we studied the quantitative variations in mit-n compatibility and the lifespan of a panel of RIAILs where difference in longevity among individual strains was a quantitative trait. These RIAILs differ from each other in many loci and a set of 1454 SNP markers spanning 98.6% of the physical length of the chromosomes had been previously genotyped^31^. The 1454 SNPs could be used to estimate the amount of HW or N2 nuclear genome in a particular RIAIL strain. We calculated the HW allele content (HAC) of each strain and determined the origin of mitochondria in each strain. For strains with the same type of mitochondria (either the HW or the N2 type), a strain with a higher value of HAC would therefore represent either a better mit-n compatibility if the strain carries the HW mitochondria or a poorer compatibility if the strain has the N2 mitochondria.

We here present experimental evidence for the novel hypothesis that the quantitative trait of aging may be in part explained by the quantitative variations in the mit-n compatibility on a genome-wide scale.

## Results

### Mitotype dependent correlation between HAC and the lifespan of *C. elegans*

**Figure 1:**
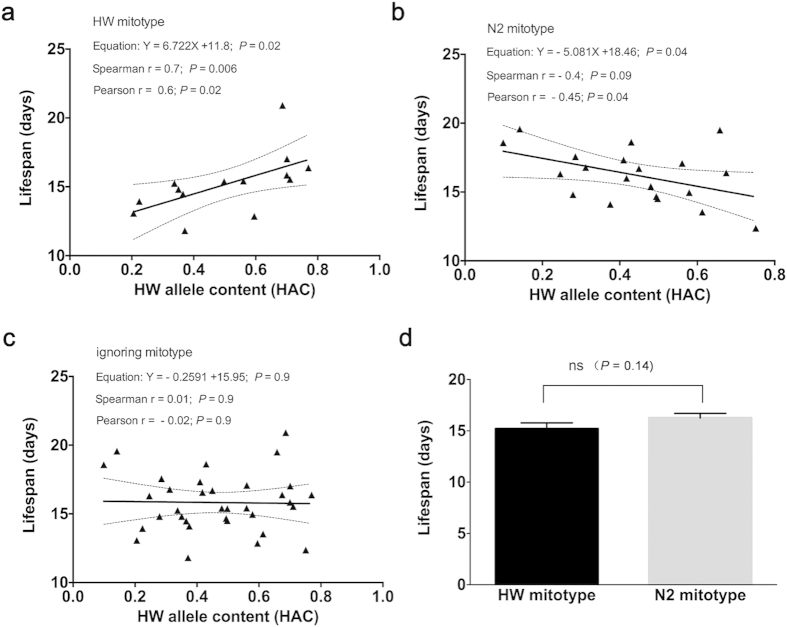
Correlation between HAC or mitotypes and lifespan in C. elegans. Correlation between HAC and lifespan in HW mitotype background (**a**) in N2 mitotype background (**b**) or ignoring the mitotype background (**c**). *P* values are shown for linear regression, Spearman, and Pearson analyses. Average lifespans in RIAIL strains with HW or N2 mitotype are shown in (**d**).

Using the set of 1454 SNPs with previously published genotype information in RIAILs, we calculated the HAC values of each strain by dividing the number of HW alleles carried by each strain by the total number of SNPs examined. We also determined the mitochondria type of each strain by PCR and found the same results as previously reported by others^31^. The HAC values and the mitochondria type of each strain were listed in Supplementary Table S1. We determined the lifespans of RIAIL strains and found a positive correlation between HAC and the lifespan of *C. elegans* in the HW mitochondria background (Fig. 1a, Supplementary Table S1). Consistently, there was an inverse correlation between HAC and the lifespan in the N2 mitotype background (*P* < 0.05 for both Pearson and linear regression analyses, Fig. 1b). We further found that there were no relationships between HAC and lifespan when ignoring the mitotype background (Fig. 1c). Also, RIAIL strains with the HW mitotype had similar or slightly shorter average lifespan than strains with the N2 mitotype (Fig. 1d). The HW CB5846 parental strain also had shorter lifespan than N2 (15.2 vs 18.0 days, Supplementary Table S1), and the average lifespans of the HW or N2 mitotypes RIAILs were similar to the HW or N2 parental strain (Fig. 1d). So, neither HAC alone nor mitotype alone could account for the combined effect of HAC and mitotype. We further tested whether just about any complex trait could be affected by mit-n compatibility by studying the foraging behaviors or food-lawn leaving rates of RIAILs using phenotype data from previous publications^33^. This trait however had no relationship with mit-n compatibility (Supplementary Fig. 1 and Supplementary Table S1). These results show a specific correlation between the degree of mit-n compatibility and lifespan.

### SNPs linked with lifespan

**Figure 2:**
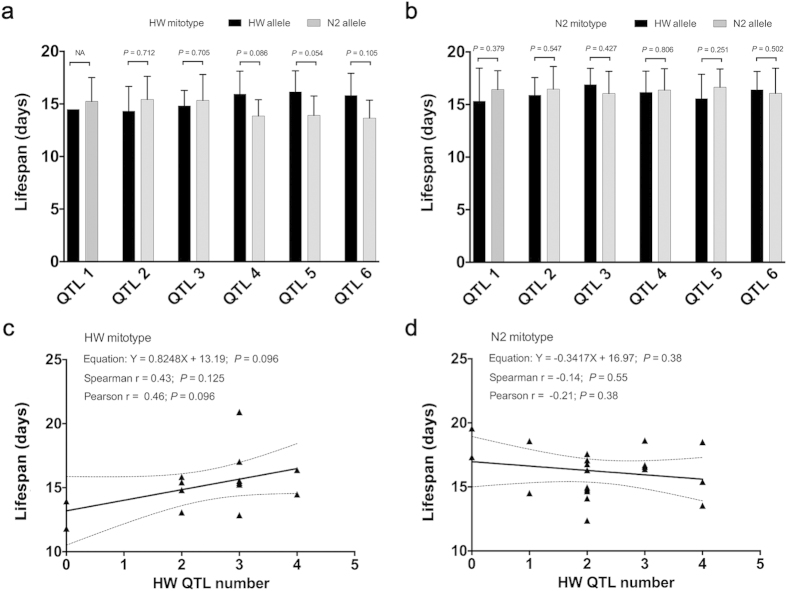
Effects on lifespans of 6 known aging linked QTLs in combination with mitotypes. Shown are average lifespan data of RIAILs with either the HW or N2 allele types of 6 aging linked QTLs and the mitotype being either HW (**a**) or N2 (**b**). The RIAILs used here all contained the HW mitochondria. The number of HW alleles of these 6 QTLs in each RIAIL was counted and correlated with lifespan in either HW mitotype RIAILs (**c**) or N2 mitotype RIAILs (**d**).

We next studied several previously identified aging-linked QTLs regions to determine whether they may account for the mit-n compatibility result here^30^. Since all HW types of these 6 QTLs are associated with reduced lifespan, a combination of the HW type QTLs with the HW mitotype would be expected to be different from the combination of N2 type QTLs with the HW mitotype. We selected the SNPs nearest to the SNPs with the highest LOD within these QTLs regions as candidate cosegregating markers (Supplementary Table S2). The result did not show a dependence of lifespans on the mitotype-QTL combinations, although two of the 6 QTLs, QTL4 and QTL5, showed slightly higher lifespan for the HW allele matched with the HW mitotype relative to that of N2 allele matched with the HW mitotype (0.05 < P < 0.1, Student’s t test, two tails, Fig. 2a–b).

These 6 loci were identified in introgression lines with N2 mitotype and mostly N2 nuclear genome (~97%)^30^. That the HW alleles of these 6 loci all have the same direction of effect (lifespan shortening) is unexpected by chance and may in fact reflect mit-n compatibility as found here. Although no significant relationship with mitotype was found in Fig. 2a–b when examined individually, such result is not unexpected because the mit-n compatibility found here is dependent on the quantity of the nuclear genome. We then studied the collective effects of these QTLs to see whether RIAILs with different numbers of the HW alleles of these 6 QTLs may show different lifespan. We counted the number of HW alleles of these 6 QTLs in each RIAILs and performed a correlation analysis with lifespan. We observed a weak trend of correlation between higher number of HW alleles with higher lifespan in HW mitotype RIAILs or with shorter lifespan in N2 mitotype RIAILs (Fig. 2c–d, Supplementary Table S2). The results in N2 mitotype worms were consistent with previous findings of lifespan shortening effects of the HW alleles in worm strains with N2 mitotype and largely N2 nuclear genomes^30^. The apparent lifespan increasing trend of these HW alleles in combination with HW mitotype is unexpected from previous work^30^, but is consistent with the relationship between mit-n compatibility and lifespan. That these 6 loci were insufficient to establish a significant correlation further supports the notion that the mit-n compatibility in lifespan is a genome wide phenomenon involving many loci.

To estimate the number of nuclear loci with a potential role in aging, we searched for SNPs that may be linked with lifespan by using the quantitative trait association option of the PLINK software^34^. Among the set of 1454 SNPs examined, we found by logistic regression test 189 SNPs linked with lifespan in the RIAILs with HW mitotype, and 134 SNPs in the RIAILs with the N2 mitotype (*P* < 0.05, Supplementary Table S3). Among these SNPs, 30 were linked with lifespan in both HW and N2 mitotypes (Supplementary Table S3). Thus, a total of 293 SNPs representing 293/1454 or 20.2% of the genome were linked with lifespan. While a multiplicity adjustment test such as the Bonferronni correction would deem all of these SNPs as insignificant, such correction is known to be overly conservative and may eliminate many true positives^35^. Among SNPs linked with lifespan in the HW mitotype, nearly half (89/189) are located on the X chromosome, whereas none is on the X for SNPs found for the N2 mitotype. This indicates some specificity here as purely chance association should not be expected to be chromosome specific. Most of the SNPs found on X do not appear to be cosegregating with npr-1 as SNPs closest to npr-1 were not found linked with lifespan. Consistent with a role of mit-n compatibility in lifespan, all of the HW alleles of SNPs linked with lifespan in HW mitotypes increased lifespan whereas the opposite was found in the N2 mitotype (Supplementary Table S3). Thus, while we cannot be certain that all these SNPs identified here are truly linked with lifespan, the high numbers found and the specific pattern of their association with mitotypes indicate a potentially large number of nuclear loci with a role in mit-n compatibility and lifespan.

### ATP related characteristics and mit-n compatibility in RIAILs

**Figure 3:**
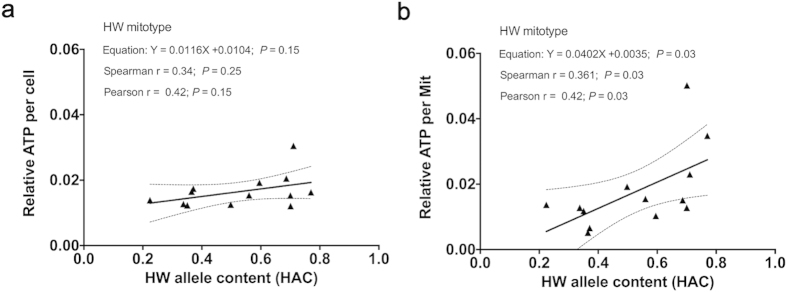
ATP content among the strains with HW mitotype. Correlation between HAC and relative ATP content per cell (**a**) and ATP content per mitochondria (**b**). All the strains used were on HW mitotype background.

To study the molecular mechanisms of the association between aging and the degree of mit-n compatibility, we examined the ATP levels of these strains to determine how well the mitochondria were functioning in producing ATP. The results did not show a correlation between the ATP levels per cell and HAC (Fig. 3a). We then determined mitochondria content (the relative copy number of mitochondrial DNA) per worm and found that the ATP levels per cell were correlated with a lower degree of mit-n compatibility (Fig. 3b).

**Figure 4:**
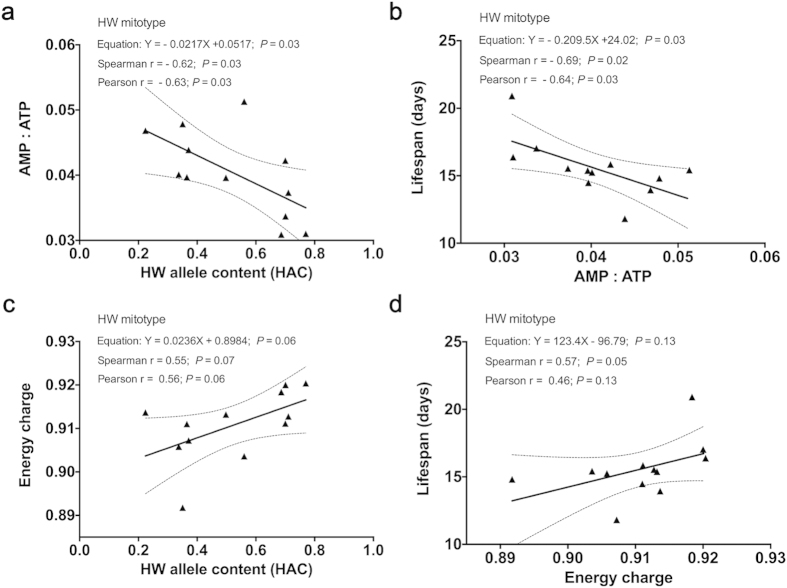
Correlation between HAC or lifespans and AMP:ATP ratio or energy charge. (**a**) Correlation between HAC and AMP:ATP ratio. (**b**) Correlation between lifespans and AMP:ATP ratio. (**c**) Correlation between HAC and energy charge. (**d**) Correlation between lifespans and energy charges. All the strains used were on the HW mitotype background.

We next determined the AMP:ATP ratio, which is known to regulate adenosine monophosphate (AMP)-activated protein kinase (AMPK) and to inversely correlate with life expectancy^36^. We found that the mean AMP:ATP ratio was inversely correlated with both HAC and mean lifespan in the HW mitotype (Fig. 4a–b).

Energy charge, representing the extent that adenine nucleotides exist as high-energy phosphates, is another measure known to be related to both mitochondria function and lifespan^37^. We calculated the energy charge of each strain, equal to (ATP + 1/2 ADP)/(ATP + ADP + AMP), and found a weak association between the mean energy charge and both HAC and mean lifespan (Fig. 4c–d). The results are consistent with a role for matched mit-n in mitochondrial functions.

### Oxidative respiration pathways in mit-n compatibility and lifespan

Genes important for mitochondrial functions have emerged as a principal group of genes affecting *C. elegans* lifespan^29^. To determine whether genes involved in mitochondrial functions may be regulated by mit-n compatibility, we studied the previously published normalized gene expression data of RIAILs in the HW mitotype background^38^. By correlating the degree of mit-n match up with gene expression profiles, we found 407 genes with their expression levels correlated with mit-n compatibility at a false discovery rate (FDR) of 5% by using the most stringent criterion according to the SAM analysis software (Supplementary Table S4)^39^. Of these 407 genes, 320 genes showed greater expression in strains with higher mit-n compatibility (Supplementary Table S4).

Using the DAVID databases^40^, we performed functional annotation of these 407 genes. There was an enrichment of genes involved in the oxidative respiration, including terms of iron, heme, monooxygenase, metalloprotein, cytochrome P450, and oxidoreductase (Supplementary Table S5). The most significantly linked biological pathway as found by Kyoto Encyclopedia of Genes and Genomes (KEGG) was related to oxidative phosphorylation, comprising 25 upregulated genes including *cyc-1*, *cco-1*, *F26E4.6*, *F57B10.14* and *W09C5.8* (Supplementary Table S6).

We also examined whether genes involved in oxidative phosphorylation may also be similarly correlated with HAC. Using a set of 82 strains, we found 136 genes associated with HAC at FDR 5% (Supplementary Table S7). Of these, only 11 genes were shared with the above 407 genes and only one, cyp-33D1, was related to oxidative phosphorylation. Thus, there was a significant enrichment of genes of the oxidative phosphorylation pathway among genes correlated with mit-n compatibility relative to those correlated with HAC (25/407 vs 1/136, P < 0.01, Fisher Exact Test, two tailed). These results suggest a role for mit-n compatibility but not HAC in the expression of genes related to oxidative phosphorylation pathway.

## Discussion

We tested the novel hypothesis that the quantitative trait of lifespan in a population could be explained in part by the degree of mit-n compatibility. By employing the novel HAC method of quantifying mit-n compatibility in *C. elegans* RIAILs, our results show a correlation between mit-n compatibility and a quantitative trait such as lifespan in worms. The genetic component of a complex quantitative trait is usually explained as a result of nuclear genome variations such as SNPs. Our results here provide evidence implicating mit-n compatibility in the quantitative nature of a complex trait.

Nuclear variations affecting the lifespans of the *C. elegans* have been well established by previous studies^30^,^41^,^42^. In particular, the HW types of 6 previously identified aging-linked QTLs show reduced lifespan^30^. Although we did not find any of these SNPs to have a significant relationship with mitotype when examined individually, we did find a consistent trend of a specific relationship between the collective effects of these 6 QTLs and mitotypes. Such a relationship implies a role for these QTLs in mit-n compatibility in addition to their previously identified role in aging that appears to be independent of mitotypes as well as of other nuclear loci. Although it is possible that the examined SNPs may not be truly cosegregating with these QTLs, it seems unlikely that such a low possibility event of no linkage despite close physical distance would occur in all 6 cases. Thus, the lifespan relationship with mit-n compatibility here may involve loci not previously identified and suggests the existence of nuclear loci which may be mitotype dependent during aging. This is consistent with our finding of lifespan association with ~20% of genome wide SNPs. Most of these lifespan linked SNPs identified here appear specific as they are consistent with a role in mit-n compatibility; the HW alleles of those SNPs linked with lifespan in HW mitotype all increased lifespan whereas those found for the N2 mitotype all decreased lifespan. That the number of SNPs involved in mit-n compatibility in lifespan is large rather than small is consistent with the high number of genes directly involved in mitochondria function (there are more than 1000 such genes and many more if one also includes those that regulate them). Future characterization and enumeration of mitochondria-related nuclear genes in worms should make it feasible to examine the next question: are these SNPs enriched in genes or genomic regions involved in mitochondria functions?

The CB4856 strain has complete mit-n matchup but its lifespan is shorter than N2 and is not longer than some RIAILs with only partial HW genome matched with HW mitotype. This indicates that higher lifespan in RIAILs with HW mitotype may involve both HW nuclear genome and N2 nuclear genome with HW genome playing a more significant role.

The HW mitochondria appears to be less potent or optimal than the N2 type as it has been shown that the variation of p.A12S amino acid in the HW mitochondria increased mitochondrial matrix oxidant burden and sensitivity to oxidative stress^26^. Our finding of a stronger mit-n epistasis in aging in the HW mitotype relative to the N2 background indicates that the effect of mit-n epistasis on mitochondrial functions may be more pronounced for less optimal forms of mitochondria, which is *a priori* expected.

We further show a correlation between the mit-n compatibility and several functional characteristics of mitochondria such as the ATP content per mitochondria, the AMP:ATP ratio, energy charge, and enrichment of genes of the oxidative phosphorylation pathway. These results are consistent with a functional effect of mit-n compatibility on mitochondria. The finding of mit-n compatibility in regulating gene expression is consistent with expectations of changes in gene expression during aging. It has also been reported that heritable regulation of gene expression becomes more polygenic in aging worms^17^,^43^.

Our results suggest that the correlation between HAC values and lifespan as found here is due to mit-n compatibility rather than HAC per se. First, the HW strains did not have higher lifespan than the N2 strains. Second, there were only 11 genes with expressions correlated with both HAC and the mit-n compatibility, and there was only one gene that was related to oxidative phosphorylation among genes correlated with HAC. In contrast, there were 25 genes with expressions correlated with the mit-n compatibility that have oxidative phosphorylation function.

The traits of RIAILs may be related to hybrid dysgenesis referring to the appearance of abnormal traits in post cross-species hybridizations. Our results suggest that poor mit-n compatibility could account for the shorter lifespan traits and other related traits in some of the hybrid strains. May such results be merely specific to the period of hybrid dysgenesis and hence not relevant after long time evolution for a well-adapted ‘normal’ species? Clearly one cannot do long term experiments to address this issue directly. However, there are good reasons and data in favor of a role for mit-n compatibility in normal organisms. First, *a priori*, a mit-n relationship can only be of three types, positive, inverse and no relationship. The only sensible choice here would be a positive relationship given the known role of over 1000 nuclear genes in mitochondrial function. Second, the evolutionary history of many normal species may involve hybridization followed by a post cross period of inbreeding and dysgenesis, e.g., ancient DNA data have refuted the long-standing notion of no hybridization between modern humans and the Neanderthals^44^,^45^. Inbreeding in humans was also common in ancient times as may be expected for small tribal societies^45^. While Neanderthal nuclear genomes have been detected in low amounts in today’s humans, no Neanderthal mtDNA could be detected despite the fact that over 30000 modern human mtDNA have been sequenced (number of mtDNAs in Genbank). Ancient DNA analysis of anatomically modern humans has shown that ancient mtDNAs of modern humans are very different from those of contemporary Neanderthals but are well within the variations of today’s humans^45^. Thus, mit-n compatibility could have played an important role in human evolution. Hybrid humans with Neanderthal mtDNA and largely modern nuclear genomes may have poorer traits relative to those with modern human mtDNA due to mit-n incompatibility and may hence have gone extinct. Thus, the results here may explain the puzzle of no trace of Neanderthal mtDNA in modern humans despite the presence of Neanderthal nuclear genomes.

Overall, our results establish a correlation between mit-n compatibility and the quantitative variations in lifespan as well as in several mitochondrial functions in a panel *C. elegans* RIAILs. Such a correlation is *a priori* expected to be a causal relationship, which could be further established by future studies.

## Materials and Methods

### Strains and media

*C. elegans* RIAILs were gifts from L. Kruglyak. *C. elegans* were cultivated at 20 °C on normal growth medium (NGM) and seeded with the *E. coli* OP50.

### HAC calculation

The 1454 SNPs with genotype data for the RIAILs were downloaded from previously published dataset^31^. The number of HW alleles of these SNPs in each RIAIL strain was counted, which was then used to divide the number 1454 to obtain the HAC value for each strain.

### Association Mapping

The PLINK software package (v1.07) with the quantitative trait association option was used to search for linked SNPs. For the additive effects of SNPs, the direction of the logistic regression coefficient represents the effect of each extra minor allele (i.e., a positive regression coefficient means that the minor allele increases lifespan mean). We did not remove SNPs in perfect linkage disequilibrium with other SNPs because all these SNPs can be used to discern the genomic extent of intervals associated with traits^31^.

### Lifespan assays

Lifespan assays were conducted on NGM agar at 20 °C as described previously^46^. The *C. elegans* grown on NGM plates (three plates, about 80 animals on each plate) with *E.coli* OP50 were scored every day for touch-provoked movement with a platinum wire; animals that failed to respond were considered dead. Each experiment was repeated 3 times. The mean lifespan was calculated from the results of three experiments.

### Assay for the origin of mitochondria

Mitochondria were isolated as previously described^47^. Two SNPs that can distinguish N2 and HW strain were determined by PCR. SNP1 is located at 2038 site in the N2 mitochondrial genome LK928807 (AGAATGATTTACGTTACC**A/T**TATTTTTTTGA TTTT, A = N2, T = HW), and SNP2 is located at 3444 site in the mitochondrial genome LK928807 (ATTTCTTTATTTAC**C/G**TTGTTTTTAACATTAT, C = N2, G = HW). The nuclear DNA was extracted from worms and amplified with SNP1 and SNP2 primers (SNP1, F, ATAACACCCTTAAATTCCTC, R, CTAACTCCCTTTCACCTTC; SNP2, F, CAACTAACGAGTTCATAAAGCAA, R, GACCTCCTCTACAAAGAAGAAATAA).

### Measurements of relative mtDNA content per cell

We used quantitative real-time PCR to determine the relative copy number of mtDNA to nuclear DNA. The copy number of mtDNA per cell was determined as follows. The 1 day old adult worms were washed three times by sterile water to remove the *E. coli* OP50. Then the washed worms were transferred to the PCR tube which had 10 μL sterile water containing 100 μg/ml proteinase K (each tube had only one worm and 20 worms were examined for each strain). The PCR tubes underwent freezing and thawing for two times (liquid nitrogen 1 min, room temperature 5 min), and was then heated at 56 °C for 15 min and at 95 °C for 10 min. The solutions were then mixed with 10 μL SYBR Green Supermix (cat #170-8882AP) and amplified with primers specific to either mtDNA (F, GTTTATGCTGCTGTAGCGTG, R, CTGTTAA AGCAAGTGGACGAG) or nuclear DNA (F, TGGAACTCTGGAGTCACACC, R, C ATCCTCCTTCATTGAACGG)^48^. Every experiment was repeated three times and the mean value was used for correlation studies.

### Assays for AMP, ADP, and ATP levels

ATP concentration in 1 day old adult worm was measured by ATP bioluminescent assay kit. The number of worms was counted (about 300 adults), and the worms were then washed 3 times with M9 buffer (22 mM KH2PO4, 42 mM Na2HPO4, 85 mM NaCl, 1 mM MgSO4) containing 0.1% Tween-20 to remove *E. coli* OP50 (all but 500 μL of M9 buffer was removed each time from the worm pellet). Worms were then lysed as described previously^49^. ATP was measured using a luciferase based assay (Sigma, product number FL-AA), using the manufacturer’s protocol. The ATP concentrations were normalized by the number of animals. All the strains of worms used in the study were examined at the same ages and each experiment was repeated 3 times. The final ATP concentration used for correlation studies was the mean of the normalized ATP concentration.

AMP, ADP, ATP was measured using HPLC as previously described^50^. About 100 to 300 worms (1 day adult) were washed three times as described above (all but 20 μL wash buffer were removed each time). The 20 μL worms were lysed in 80 μL of ice-cold 8% (v/v) HClO4 immediately followed by three intervals of 30 sec sonication and 30 sec on ice. The solution was neutralized with 1 N K_2_CO_3_ and centrifuged briefly, and the supernatant was passed through a 0.2-μm filter (Nanosep), and subjected to reversed phase chromatography using a Hypersil ODS2 250 × 4.6 mm 5-μm column. Nucleotides were detected at 260 nm with a Shimazdu SPD-6AV detector. Peak areas were measured using Peak Explorer software.

### Statistical methods

Spearman, Pearson, and linear regression analyses were performed using GraphPad Prism5. Normalized gene expression data for the RIAILs were obtained from published datasets^38^. The correlation between gene expression and HAC was analyzed using the Significance Analysis of Microarrays (SAM) software with 1,000 sample permutations. SAM uses permutations to estimate the false discovery rate (FDR) and an adjustable threshold allows for control of the FDR. SAM adopts q-value as the lowest FDR at which the gene is called significant. We used the most stringent criteria as defined by SAM to call a gene significant, which are treating data as quantitative type, 1000 permutations, 5% FDR, and KNN value 10 (K-nearest neighbor).

DAVID bioinformatics resources (DAVID) consists of an integrated biological knowledgebase and analytic tools aiming at systematically extracting biological meaning from large gene/protein lists^40^. The functional classification tool of DAVID generates a gene-to-gene similarity matrix based on shared functional annotation using over 75,000 terms from 14 functional annotation sources. The novel clustering algorithms classifies highly related genes into functionally related groups. The well-known KEGG pathway can also be predicted by the DAVID tool. The detailed protocol for using DAVID can be found at http://david.abcc.ncifcrf.gov or a previous description^40^.

## Additional Information

**How to cite this article**: Zhu, Z. *et al.* Compatibility between mitochondrial and nuclear genomes correlates with the quantitative trait of lifespan in *Caenorhabditis elegans.* *Sci. Rep.* **5**, 17303; doi: 10.1038/srep17303 (2015).

## Supplementary Material

Supplementary Information

Supplementary Dateset 1

Supplementary Dateset 2

Supplementary Dateset 3

Supplementary Dateset 4

## References

[b1] Sebastiani P. *et al.* Meta-analysis of genetic variants associated with human exceptional longevity. Aging (Albany NY) 5, 653–661 (2013). 24244950 10.18632/aging.100594 PMC3808698

[b2] Deelen J., Beekman M., Capri M., Franceschi C. & Slagboom P. E. Identifying the genomic determinants of aging and longevity in human population studies: progress and challenges. BIOESSAYS 35, 386–396 (2013). 23423909 10.1002/bies.201200148 PMC3633240

[b3] Beekman M. *et al.* Genome-wide linkage analysis for human longevity: Genetics of Healthy Aging Study. Aging Cell 12, 184–193 (2013). 23286790 10.1111/acel.12039 PMC3725963

[b4] Brand M. D. The role of mitochondria in longevity and healthspan. Longev Healthspan 3, 7 (2014). 24855560 10.1186/2046-2395-3-7 PMC4030464

[b5] Nabholz B., Glemin S. & Galtier N. Strong variations of mitochondrial mutation rate across mammals–the longevity hypothesis. Mol Biol Evol 25 120–130 (2008). 17998254 10.1093/molbev/msm248

[b6] Li L. *et al.* Mitochondrial genomes and exceptional longevity in a Chinese population: the Rugao longevity study. Age 37, 1 1–10 (2015). 10.1007/s11357-015-9750-8 PMC4322039 25666573

[b7] De Benedictis G. *et al.* Mitochondrial DNA inherited variants are associated with successful aging and longevity in humans. Faseb J 13, 1532–1536 (1999). 10463944 10.1096/fasebj.13.12.1532

[b8] HARMAN D., Aging: a theory based on free radical and radiation chemistry. J Gerontol 11, 298–300 (1956). 13332224 10.1093/geronj/11.3.298

[b9] Schapira A. H., Mitochondrial diseases. Lancet 379, 1825–1834 (2012). 22482939 10.1016/S0140-6736(11)61305-6

[b10] Balaban R. S., Nemoto S. & Finkel T., Mitochondria, oxidants, and aging. Cell 120, 483–495 (2005). 15734681 10.1016/j.cell.2005.02.001

[b11] Muftuoglu M. *et al.* Mitochondrial complex I and IV activities in leukocytes from patients with parkin mutations. Mov Disord 19, 544–548 (2004). 15133818 10.1002/mds.10695

[b12] Barja G. Endogenous oxidative stress: relationship to aging, longevity and caloric restriction. Ageing Res Rev 1, 397–411 (2002). 12067594 10.1016/s1568-1637(02)00008-9

[b13] Lee S. J., Hwang A. B. & Kenyon C. Inhibition of respiration extends C. elegans life span via reactive oxygen species that increase HIF-1 activity. Curr Biol 20, 2131–2136 (2010). 21093262 10.1016/j.cub.2010.10.057 PMC3058811

[b14] Heidler T., Hartwig K., Daniel H. & Wenzel U. Caenorhabditis elegans lifespan extension caused by treatment with an orally active ROS-generator is dependent on DAF-16 and SIR-2.1. Biogerontology 11, 183–195 (2010). 19597959 10.1007/s10522-009-9239-x

[b15] Yang W. & Hekimi S. A mitochondrial superoxide signal triggers increased longevity in Caenorhabditis elegans. Plos Biol 8, e1000556 (2010). 21151885 10.1371/journal.pbio.1000556 PMC2998438

[b16] Jensen M. B. & Jasper H. Mitochondrial proteostasis in the control of aging and longevity. Cell Metab 20, 214–225 (2014). 24930971 10.1016/j.cmet.2014.05.006 PMC4274350

[b17] Vinuela A., Snoek L. B., Riksen J. A. & Kammenga J. E. Genome-wide gene expression regulation as a function of genotype and age in C. elegans. Genome Res 20, 929–937 (2010). 20488933 10.1101/gr.102160.109 PMC2892094

[b18] Dowling D. K., Abiega K. C. & Arnqvist G. Temperature-specific outcomes of cytoplasmic-nuclear interactions on egg-to-adult development time in seed beetles. Evolution 61, 194–201 (2007). 17300438 10.1111/j.1558-5646.2007.00016.x

[b19] Meiklejohn C. D. *et al.* An Incompatibility between a mitochondrial tRNA and its nuclear-encoded tRNA synthetase compromises development and fitness in Drosophila. Plos Genet 9, e1003238 (2013). 23382693 10.1371/journal.pgen.1003238 PMC3561102

[b20] Zhu C. T., Ingelmo P. & Rand D. M. GxGxE for lifespan in Drosophila: mitochondrial, nuclear, and dietary interactions that modify longevity. Plos Genet 10, e1004354 (2014). 24832080 10.1371/journal.pgen.1004354 PMC4022469

[b21] Paliwal S., Fiumera A. C. & Fiumera H. L. Mitochondrial-nuclear epistasis contributes to phenotypic variation and coadaptation in natural isolates of Saccharomyces cerevisiae. Genetics, 198 1251–1265 (2014). 25164882 10.1534/genetics.114.168575 PMC4224164

[b22] Bar-Yaacov D., Blumberg A. & Mishmar D. Mitochondrial-nuclear co-evolution and its effects on OXPHOS activity and regulation. Biochim Biophys Acta 1819, 1107–1111 (2012). 22044624 10.1016/j.bbagrm.2011.10.008

[b23] Osada N. & Akashi H. Mitochondrial-nuclear interactions and accelerated compensatory evolution: evidence from the primate cytochrome C oxidase complex. Mol Biol Evol 29, 337–346 (2012). 21890478 10.1093/molbev/msr211

[b24] Levin L., Blumberg A., Barshad G. & Mishmar D. Mito-nuclear co-evolution: the positive and negative sides of functional ancient mutations. Front Genet 5 448 (2014). 25566330 10.3389/fgene.2014.00448 PMC4274989

[b25] Gershoni M. *et al.* Disrupting mitochondrial-nuclear coevolution affects OXPHOS complex I integrity and impacts human health. Genome Biol Evol 6, 2665–2680 (2014). 25245408 10.1093/gbe/evu208 PMC4224335

[b26] Dingley S. D. *et al.* Mitochondrial DNA Variant in COX1 Subunit Significantly Alters Energy Metabolism of Geographically Divergent Wild Isolates in Caenorhabditis elegans. J Mol Biol 426, 2199–2216 (2014). 24534730 10.1016/j.jmb.2014.02.009 PMC4067970

[b27] Troemel E. R. *et al.* p38 MAPK regulates expression of immune response genes and contributes to longevity in C. elegans. Plos Genet 2, e183 (2006). 17096597 10.1371/journal.pgen.0020183 PMC1635533

[b28] Murphy C. T. *et al.* Genes that act downstream of DAF-16 to influence the lifespan of Caenorhabditis elegans. Nature 424 277–283 (2003). 12845331 10.1038/nature01789

[b29] Lee S. S. *et al.* A systematic RNAi screen identifies a critical role for mitochondria in C. elegans longevity. Nat Genet 33, 40–48 (2003). 12447374 10.1038/ng1056

[b30] Doroszuk A., Snoek L. B., Fradin E., Riksen J. & Kammenga J. A genome-wide library of CB4856/N2 introgression lines of Caenorhabditis elegans. Nucleic Acids Res 37, e110 (2009). 19542186 10.1093/nar/gkp528 PMC2760803

[b31] Rockman M. V. & Kruglyak L. Recombinational landscape and population genomics of Caenorhabditis elegans. Plos Genet 5, e1000419 (2009). 19283065 10.1371/journal.pgen.1000419 PMC2652117

[b32] Solorzano E. *et al.* Shifting patterns of natural variation in the nuclear genome of caenorhabditis elegans. Bmc Evol Biol 11, 168 (2011). 21679441 10.1186/1471-2148-11-168 PMC3151237

[b33] Bendesky A., Tsunozaki M., Rockman M. V., Kruglyak L. & Bargmann C. I. Catecholamine receptor polymorphisms affect decision-making in C. elegans. Nature 472, 313–318 (2011). 21412235 10.1038/nature09821 PMC3154120

[b34] Purcell S. *et al.* PLINK: a tool set for whole-genome association and population-based linkage analyses. Am J Hum Genet 81, 559–575 (2007). 17701901 10.1086/519795 PMC1950838

[b35] Perneger T. V., What’s wrong with Bonferroni adjustments. BMJ 316, 1236–1238 (1998). 9553006 10.1136/bmj.316.7139.1236 PMC1112991

[b36] Apfeld J., O’Connor G., McDonagh T., DiStefano P. S. & Curtis R. The AMP-activated protein kinase AAK-2 links energy levels and insulin-like signals to lifespan in C. elegans. Genes Dev 18, 3004–3009 (2004). 15574588 10.1101/gad.1255404 PMC535911

[b37] Curtis R., O’Connor G. & DiStefano P. S. Aging networks in Caenorhabditis elegans: AMP-activated protein kinase (aak-2) links multiple aging and metabolism pathways. Aging Cell 5, 119–126 (2006). 16626391 10.1111/j.1474-9726.2006.00205.x

[b38] Rockman M. V., Skrovanek S. S. & Kruglyak L. Selection at linked sites shapes heritable phenotypic variation in C. elegans. Science 330 372–376 (2010). 20947766 10.1126/science.1194208 PMC3138179

[b39] Tusher V. G., Tibshirani R. & Chu G. Significance analysis of microarrays applied to the ionizing radiation response. Proc Natl Acad Sci USA 98, 5116–5121 (2001). 11309499 10.1073/pnas.091062498 PMC33173

[b40] Huang D. W., Sherman B. T. & Lempicki R. A. Systematic and integrative analysis of large gene lists using DAVID bioinformatics resources. Nat Protoc 4, 44–57 (2009). 19131956 10.1038/nprot.2008.211

[b41] Johnson T. E. *et al.* Longevity genes in the nematode Caenorhabditis elegans also mediate increased resistance to stress and prevent disease. J Inherit Metab Dis 25, 197–206 (2002). 12137228 10.1023/a:1015677828407

[b42] Hamilton B. *et al.* A systematic RNAi screen for longevity genes in C. elegans. Genes Dev 19, 1544–1555 (2005). 15998808 10.1101/gad.1308205 PMC1172061

[b43] Vinuela A., Snoek L. B., Riksen J. A. & Kammenga J. E. Aging Uncouples Heritability and Expression-QTL in Caenorhabditis elegans. G3 (Bethesda) 2, 597–605 (2012). 22670229 10.1534/g3.112.002212 PMC3362942

[b44] Fu Q. *et al.* An early modern human from Romania with a recent Neanderthal ancestor. Nature 524, 216–219 (2015). 26098372 10.1038/nature14558 PMC4537386

[b45] Pr U, Fer K. *et al.* The complete genome sequence of a Neanderthal from the Altai Mountains. Nature 505, 43–49 (2014). 24352235 10.1038/nature12886 PMC4031459

[b46] Hansen M., Hsu A. L., Dillin A. & Kenyon C. New genes tied to endocrine, metabolic, and dietary regulation of lifespan from a Caenorhabditis elegans genomic RNAi screen. Plos Genet 1, 119–128 (2005). 16103914 10.1371/journal.pgen.0010017 PMC1183531

[b47] Jonassen T., Marbois B. N., Faull K. F., Clarke C. F. & Larsen P. L. Development and fertility in Caenorhabditis elegans clk-1 mutants depend upon transport of dietary coenzyme Q8 to mitochondria. J Biol Chem 277, 45020–45027 (2002). 12324451 10.1074/jbc.M204758200

[b48] Artal-Sanz M. & Tavernarakis N. Prohibitin couples diapause signalling to mitochondrial metabolism during ageing in C. elegans. Nature 461, 793–797 (2009). 19812672 10.1038/nature08466

[b49] Takahiro T., Kenji N., Hirohisa S., Masatomo M. & Ayako O. Co-operative function and mutual stabilization of the half ATP-binding cassette transporters HAF-4 and HAF-9 in Caenorhabditis elegans. Biochem J 452, 467–475 (2013). 23458156 10.1042/BJ20130115

[b50] Stocchi V. *et al.*, Simultaneous extraction and reverse-phase high-performance liquid chromatographic determination of adenine and pyridine nucleotides in human red blood cells. Anal Biochem 146, 118–124 (1985). 3993925 10.1016/0003-2697(85)90405-1

