# Supplementary material for: Compatibility between mitochondrial and nuclear genomes correlates with the quantitative trait of lifespan in Caenorhabditis elegans
Source: Sci Rep. 2015 Nov 25;5:17303. doi: 10.1038/srep17303 (PMC4658563; doi:10.1038/srep17303)
Supplement: Supplementary Information [file srep17303-s1.pdf]

Supplementary information

Compatibility between mitochondrial and nuclear genomes correlates with the quantitative trait of lifespan in *Caenorhabditis elegans*

Zuobin Zhu, Qing Lu, Fangfang Zeng, Junjing Wang and Shi Huang*

State Key Laboratory of Medical Genetics, School of Life Sciences, Xiangya Medical School, Central South University. 110 Xiangya Road, Changsha, Hunan, 410078, China

*Corresponding author, huangshi@sklmg.edu.cn

Supplementary Fig. S1. Correlations between food lawn leaving events and HAC of RIAILs. Correlations between leaving events and HAC in HW mitotype (**a and c**) or N2 mitotype (**b** and **d**). RIAILs with HW npr-1 genotype (**a** and **b**) or N2 npr-1 genotype(**c** and **d**) were studied separately because npr-1 polymorphism is a known modifier of the food-lawn leaving events trait. Data on leaving events were from previous publications[1].

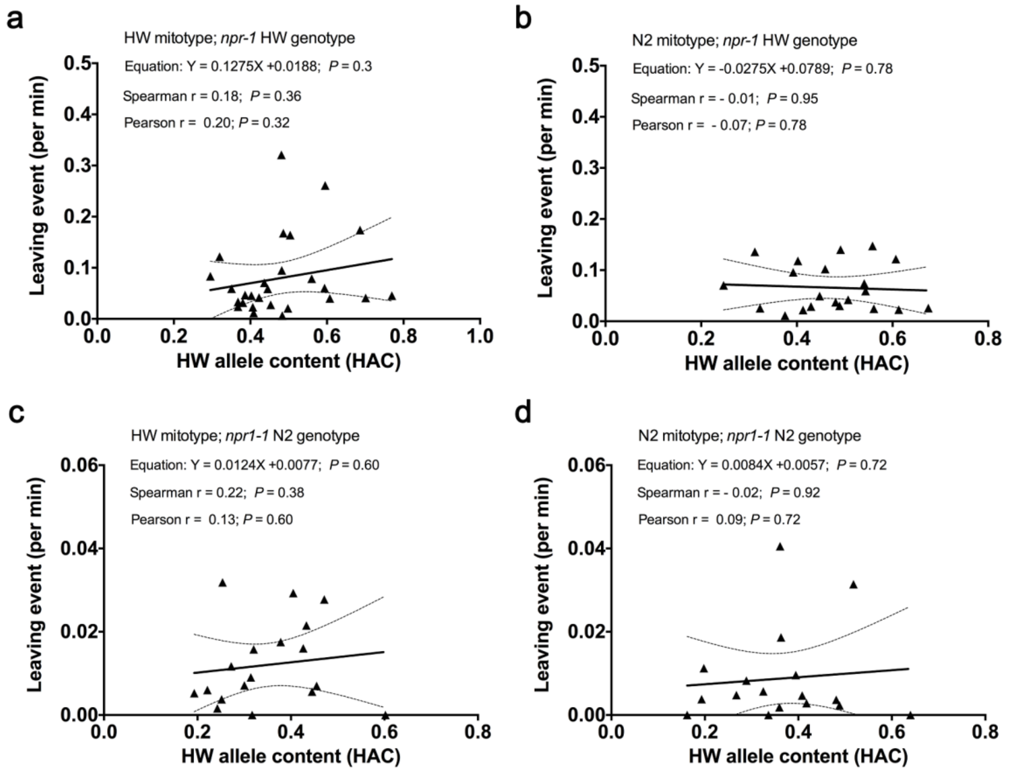

Supplementary Table S1. **Experimental data of each RIAIL strain used in this study**. (in a separate document).

Supplementary Table S2. SNPs cosegregating with the 6 QTLs linked with life span of *C. elegans*.

			CHR	1	1	2	3	4	X
			Distance to QTL	-2035	-40063	4034	7	-37658	-11112
			Marker	CE1-244	UCE1-1602	CE2-225	CE3-179	UCE4-817	UCE6-952
RIAILs	Mitotype	HAC	lifespan	QTL 1	QTL 2	QTL 3	QTL 4	QTL 5	QTL 6
QX167	CB	0.7698	12.86	N2	HW	HW	N2	N2	HW
QX54	CB	0.3710	13.07	N2	HW	N2	HW	N2	N2
QX58	CB	0.3652	17.03	N2	HW	N2	HW	N2	HW
QX133	CB	0.3508	16.38	N2	N2	HW	HW	HW	HW
QX151	CB	0.5952	14.81	N2	N2	N2	HW	N2	HW
QX204	CB	0.6864	15.25	N2	N2	HW	N2	HW	HW
QX150	CB	0.7105	14.48	HW	N2	N2	HW	HW	HW
QX179	CB	0.4979	20.91	N2	N2	N2	HW	HW	HW
QX180	CB	0.3375	15.54	N2	N2	N2	HW	HW	HW
QX198	CB	0.7003	15.38	N2	N2	N2	HW	HW	HW
QX206	CB	0.2241	15.84	N2	N2	N2	HW	HW	N2
QX136	CB	0.2055	11.81	N2	N2	N2	N2	N2	N2
QX25	CB	0.7010	13.94	N2	N2	N2	N2	N2	N2
QX65	CB	0.5601	15.42	N2	N2	N2	N2	HW	HW
QX117	N2	0.2460	16.31	N2	N2	HW	N2	N2	HW
QX12	N2	0.4296	18.62	HW	N2	N2	HW	N2	HW
QX127	N2	0.6747	16.39	N2	HW	N2	N2	HW	HW
QX13	N2	0.5608	17.08	N2	N2	N2	HW	N2	HW
QX138	N2	0.2852	17.57	N2	N2	N2	HW	N2	HW
QX159	N2	0.4495	16.7	N2	HW	HW	N2	N2	HW
QX164	N2	0.2790	14.81	N2	HW	N2	N2	N2	HW
QX194	N2	0.5794	14.96	HW	N2	N2	N2	HW	N2
QX215	N2	0.4976	14.51	N2	N2	N2	HW	N2	N2
QX218	N2	0.7512	12.37	HW	N2	N2	N2	HW	N2
QX226	N2	0.4801	15.4	N2	N2	HW	HW	HW	HW
QX237	N2	0.6584	19.5	N2	N2	HW	HW	HW	HW
QX31	N2	0.3120	16.78	N2	N2	N2	N2	HW	HW
QX33	N2	0.0990	18.58	N2	HW	N2	N2	N2	N2
QX40	N2	0.6131	13.54	N2	HW	N2	HW	HW	HW
QX43	N2	0.4175	16.56	N2	HW	HW	HW	N2	N2
QX67	N2	0.4096	17.33	N2	N2	N2	N2	N2	N2
QX72	N2	0.4942	14.67	N2	HW	N2	HW	N2	N2
QX91	N2	0.3753	14.1	N2	N2	N2	HW	N2	HW
QX97	N2	0.1416	19.57	N2	N2	N2	N2	N2	N2

Supplementary Table S3. SNPs associated with lifespan of RIAILs. (in a separate document)

Supplementary Table S4. **Genes linked with HAC in strains with the HW mitotype.** (in a separate document)

Supplementary Table S5. **Functional annotation clustering of oxidative respiration.**

Category	Term	Count	P-Value
SP_PIR_KEYWORDS	iron	9	9.50E-17
SP_PIR_KEYWORDS	heme	8	2.60E-15
SP_PIR_KEYWORDS	Monooxygenase	7	1.90E-13
SP_PIR_KEYWORDS	metalloprotein	7	3.00E-13
INTERPRO	Cytochrome P450, C-terminal region	7	5.80E-13
INTERPRO	Cytochrome P450, E-class, group I	7	6.30E-13
INTERPRO	Cytochrome P450	7	7.40E-13
GOTERM_MF_FAT	iron ion binding	9	5.00E-12
GOTERM_MF_FAT	heme binding	8	1.50E-11
GOTERM_MF_FAT	tetrapyrrole binding	8	1.70E-11
GOTERM_MF_FAT	electron carrier activity	8	1.90E-10
INTERPRO	Cytochrome P450, conserved site	6	2.40E-10
SP_PIR_KEYWORDS	oxidoreductase	7	3.10E-10

GOTERM_BP_FAT	oxidation reduction	7	8.70E-08
COG_ONTOLOGY	Secondary metabolites biosynthesis, transport, and catabolism	7	1.20E-07
SP_PIR_KEYWORDS	metal-binding	7	5.30E-07
PIR_SUPERFAMILY	PIRSF000045:cytochrome P450 CYP2D6	4	4.10E-06
GOTERM_MF_FAT	transition metal ion binding	9	9.00E-06
GOTERM_MF_FAT	metal ion binding	9	4.40E-05
GOTERM_MF_FAT	cation binding	9	5.40E-05
GOTERM_MF_FAT	ion binding	9	5.50E-05
GOTERM_BP_FAT	response to xenobiotic stimulus	2	5.30E-03

Supplementary Table S6. **Genes related to oxidative phosphorylation.**

Gene name
cyc-1
cco-1
F26E4.6
F57B10.14
W09C5.8
cyp-34A9
cyp-33D1
ZK1240.1
Y39A1A.22
asd-2
flp-2
ZK899.2
cyp-29A2
cyp-14A2
cyp-34A10
ins-33
rpl-39
ptr-11

ceh-32
F01F1.3
T26C5.5
F14B8.4
sqv-8
mtm-3
T25G3.3

Supplementary Table S7. Genes linked with HAC in 82 strains without regard to mitotypes. (in a separated document)

References:

1. Bendesky, A., Tsunozaki, M., Rockman, M. V., Kruglyak, L. & Bargmann, C. I., Catecholamine receptor polymorphisms affect decision-making in C. elegans. *NATURE* **472** 313 (2011).
2. Doroszuk, A., Snoek, L. B., Fradin, E., Riksen, J. & Kammenga, J., A genome-wide library of CB4856/N2 introgression lines of Caenorhabditis elegans. *NUCLEIC ACIDS RES* **37** e110 (2009).
